# Current and Emerging Pharmacotherapy for Fibromyalgia

**DOI:** 10.1155/2020/6541798

**Published:** 2020-02-11

**Authors:** Roie Tzadok, Jacob N. Ablin

**Affiliations:** Department of Internal Medicine H, Tel Aviv Sourasky Medical Centre and Sackler School of Medicine, Tel Aviv University, Tel Aviv, Israel

## Abstract

*Introduction*. Fibromyalgia syndrome (FMS) is a pain disorder with an estimated prevalence of 1–5%. It is associated with a variety of somatic and psychological disorders. Its exact pathogenesis is still unclear but is involved with neural oversensitization and decreased conditioned pain modulation (CPM), combined with cognitive dysfunction, memory impairment, and altered information processing. Connectivity between brain areas involved in pain processing, alertness, and cognition is increased in the syndrome, making its pharmacologic therapy complex. Only three drugs, pregabalin, duloxetine, and milnacipran are currently FDA-approved for FM treatment, but many other agents have been tested over the years, with varying efficacy. *Areas Covered*. The purpose of this review is to summarize current clinical experience with different pharmacologic treatments used for fibromyalgia and introduce future perspectives in developing therapies. *Expert Opinion*. Future insights into the fields of cannabinoid and opioid research, as well as an integrative approach towards the incorporation of genetics and functional imaging combined with additional fields of research relevant towards the study of complex CNS disorders, are likely to lead to new developments of novel tailor-made treatments for FMS patients.

## 1. Introduction

Fibromyalgia syndrome (FMS) is a common pain disorder, characterized by chronic generalized pain, with a population-estimated prevalence of 1–5% [1]. The disorder is also associated with a variety of somatic and psychological disorders, including fatigue, sleep disturbances, stiffness, anxiety, and cognitive dysfunction [2].

Over the past couple of decades, extensive research has been devoted towards the endeavor of developing pharmacological solutions for the fibromyalgia spectrum. This reality is well expected in view of the overwhelming economic burden posed by chronic pain in general and fibromyalgia in particular. These efforts have led to three medications gaining FDA approval for the fibromyalgia indication. Nonetheless, while reviewing the progress made in this area, it must be acknowledged that pharmacological treatment has been met, in general, with rather modest rates of success in this area. Real-life data published over recent years [3], together with the clinical experience of physicians dealing with this group of patients, all indicate that only a minority of fibromyalgia patients continue taking medications for more than a short period of time due to either lack of efficacy, side effects, or both. Current guidelines published regarding the treatment of fibromyalgia unanimously advocate a multidisciplinary approach, combining pharmacological treatment with complimentary modalities, including cognitive behavioral therapy (CBT), aerobic and strengthening physical training, and even meditative movement therapies [4–7].

The strikingly modest progress in this field, as manifested by the surprisingly low compliance of patients, attests to the great complexity of the mechanisms of chronic pain within the central nervous system. Despite the growing insight, through functional neuroimaging and similar modalities, into the neuroscience of pain, we are yet at the beginning. It appears that much greater insight will be necessary before a truly rational and effective pharmacological solution for fibromyalgia (and similar conditions) will be at hand.

The purpose of the present review is to provide an updated perspective of the pharmacological alternatives available for the treatment of FMS.  Table 1 summarizes the current clinical evidence regarding effective treatments for FMS.

## 2. FMS Pathophysiology

Assumed to be of neurogenic origin, both hyperalgesia and allodynia are usually found [37]. However, the exact pathogenesis of FMS is still unknown. With CNS involvement being a key element, neural oversensitization (or “central sensitization”) is suggested to be the main pathophysiological change [37–39], meaning the CNS interprets benign stimulations as unpleasant. This principal was demonstrated by an increased CNS response to stimulation and decreased conditioned pain modulation (CPM). CPM is a neural process of sensitization modulation, which involves activation of specific neurotransmitters including serotonin and noradrenaline. Thus, medications that modulate levels of these neurotransmitters in the CNS have the potential to improve CPM and reduce central sensitization [40, 41].

Other neurotransmitters, including GABA, cannabinoid receptors, substance P, NGF, and opioid receptors participate in this complex modulation of pain transmission [42], therefore functioning as optional therapeutic targets. There is also evidence that glial cells may play a role in maintaining central sensitization and contribute to chronic pain production by producing IL-6, IL-8, and other cytokines, which are found to be at high levels in FMS patients' sera [43].

Comorbid conditions, including mood disorders, anxiety, headaches, irritable bowel syndrome, sleep disturbances, and chronic fatigue syndrome, are found in a large percentage of FMS patients [2, 44, 45]. Accordingly, medications which improve sleep disorders, as well as those that improve daytime alertness, could be useful for the management of FMS. From a neurobiological perspective, sensory, affective, and cognitive centers within the brain interact in producing the final pain experience. Indeed, increased connectivity between different brain areas is a known phenomenon in FMS [46]. Increased connectivity has also been demonstrated between various other areas participating in pain processing, alertness, and cognition [46]. By perceiving brain function in general, and specifically the pathophysiology of FMS, new targets for medication development for the syndrome can be found [47].

## 3. Pharmacotherapeutic Agents Used for FMS

Only three drugs, pregabalin (a gabapentinoid that acts by blocking calcium channels), duloxetine, and milnacipran (both are serotonin-noradrenaline reuptake inhibitors), have been approved for use in the treatment of FMS by the FDA. However, other types of antidepressants are used for the treatment of various chronic pain syndromes, including FMS, with varying levels of evidence regarding their efficacy.

### 3.1. Tricyclic Antidepressants (TCAs)

Although different TCAs have been used in the treatment of chronic pain, the largest body of evidence on therapeutic utility in FMS exists regarding amitriptyline. It is recommended by all various clinical practice guidelines [8–10, 15].

Amitriptyline was found to reduce the FIQ questionnaire results from baseline to endpoint by over 30% [48–50]. It was found to improve pain, fatigue, sleep, and quality of life [11].

### 3.2. Serotonin-Noradrenaline Reuptake Inhibitors (SNRIs)

Various clinical trials published evaluating duloxetine showed a significant improvement in FMS-associated pain and depressive symptoms [11]. The vast majority of clinical trials evaluating milnacipran have shown a significant improvement in pain levels, in addition to fatigue. Results regarding improvement in psychological effects of FMS (namely, depression) are more controversial [11].

A meta-analysis reviewing five different studies regarding duloxetine and five different studies regarding and milnacipran showed that these drugs had positive effects on pain and patient-perceived clinical improvement [12].

Selective noradrenaline reuptake inhibitors (NRIs), such as reboxetine and its enantiomer esreboxetine, were also suggested as possible treatments for FMS, while the body of evidence regarding the efficacy of reboxetine is rather sporadic and is based mostly on case reports [13], esreboxetine was shown to reduce pain, fatigue, and improve overall quality of life in a randomized, double-blind, placebo-controlled trial [14]. No other trials regarding it were published.

### 3.3. Selective Serotonin Reuptake Inhibitors (SSRIs)

Among the SSRIs investigated for the treatment of FMS were citalopram, escitalopram, fluoxetine, paroxetine, and sertraline. Despite the theoretical assumption, that the combined inhibition of serotonin and noradrenaline is more efficacious than selective serotonin augmentation vis-à-vis the inhibition of pain [51], the use of this class of drugs is recommended in practice guidelines [8–10, 15]. According to the results of a meta-analysis performed by Häuser et al., SSRIs improve pain, depression, and overall quality of life, but to a small extent. The effect size for improvement in sleep disorders was found to be nonsubstantial [16].

### 3.4. Cyclobenzaprine

Cyclobenzaprine is a 5-HT2 receptor blocker, which acts on a subfamily of serotonin receptors, and causes muscle relaxation. It resembles amitriptyline in structure and is commonly used in FMS patients. A systematic review of the literature reported that it has a moderate benefit in improving sleep disturbances and only a mild improvement in pain [17]. Moldofsky et al. have previously shown that bedtime very low doses of cyclobenzaprine were shown to significantly improve pain and sleep in patients with a specific sleep architecture [18]. A sublingual formulation of low-dose cyclobenzaprine (TNX-102SL, 2.8 mg) has been reported to improve nonrestorative sleep in FMS patients [19]; however, this formulation has subsequently failed to reach primary pain-related endpoints, and its development has been stopped.

### 3.5. Mirtazapine

Mirtazapine is an atypical antidepressant with noradrenergic and specific serotonergic activity. It is not licensed for use in FMS. A meta-analysis held by Welsch et al. did not find the drug effective for pain relief in FMS nor for any other associated mental or functional symptoms related to it (depression, sleep problems, fatigue, etc.) [52].

### 3.6. Gabapentinoids

The two main members of this family of drugs, pregabalin and gabapentin, act by binding to the alpha2delta subunit of voltage-gated calcium channels in the CNS. Originally used as anticonvulsants, they are currently mainly used for the treatment of chronic pain [11]. Pregabalin has FDA approval for the treatment of FMS, and its use is recommended in guidelines [8–10, 15]. A series of placebo-controlled clinical trials showed pregabalin to improve pain and sleep disturbances. However, compared to placebo, it was not found to significantly improve complaints of fatigue in some trials, and none of the trials indicated any improvement in depressive symptoms [20, 21, 53]. A meta-analysis of randomized controlled trials regarding both pregabalin and gabapentin further emphasized their effect in improving pain, fatigue, sleep, and overall quality of life, in addition to their lack of effect on depressive symptoms and relatively nonsubstantial effect on anxiety [22].

Lacosamide is another anticonvulsant that has been evaluated as a therapeutic modality for neuropathic pain. In animal models, it effectively reduced muscular hyperalgesia and was more effective than gabapentin and pregabalin [54]. It was also found effective in a placebo-controlled, double-blind trial for treating neuropathic pain [55]. Lacosamide has been tested for the treatment of FMS in a randomized double-blind trial including 160 patients randomized for either the drug or placebo. No significant effect was demonstrated on major endpoints (pain, sleep, and cognitive function), and therefore, there is currently no clear evidence regarding the efficacy of this medication in FMS [23]. A newer gabapentinoid, mirogabalin (DS-5565) was shown to have analgesic qualities in animal models of both central and peripheral neuropathies [56], while approved for the treatment of peripheral neuropathy in Japan, its development as a treatment modality for FMS in the USA and Europe was stopped after it failed in meeting primary endpoints in phase 3 trials [57]; three 13-week randomized, double-blind studies comparing both placebo and pregabalin to two different daily doses of mirogabalin, failed to show a reduction in the average daily worse pain score (ADPS) in the mirogabalin arm [58].

Kim et al. demonstrated that low-to-moderate alcohol consumption was associated with an improvement in FMS symptoms, namely, pain level, physical and social function, general health perception, and general quality of life. The same association was not observed in heavy drinkers [59]. It was assumed that the effect might be centrally mediated through ethanol enhancement of GABA release in the CNS [60, 61].

### 3.7. Opioids

Endogenous descending antinociceptive activity is postulated to be reduced in FMS. In humans, such two descending pain inhibitory pathways exist: the noradrenaline/serotonin-mediated pathway and the opioid-mediated one [62]. Baraniuk et al. suggested an excess of endogenous opioids in FMS [63]. Following these data, Harris et al. used positron emission tomography (PET) technology to show that the availability of µ-opioid receptors in FMS patients is reduced in certain areas of the brain, possibly as a result of receptor downregulation secondary to their increased levels. Reduced availability was inversely correlated with clinical pain ratings [64]. Following these findings, naltrexone, a competitive opioid receptor antagonist, was proposed as potential new means of treating chronic pain. The beneficial effect of naltrexone on fibromyalgia symptoms was shown by Youner and Mackey in a pilot study in 2009 [24], with a randomized controlled trial published in 2013, finding it to be superior to placebo in reducing pain and associated depressive symptoms [25].

Apart from its opioid receptor antagonist activities, naltrexone also has antagonist activity to other nonopioid receptors (toll-like receptor 4) expressed on activated microglia cells, which are specialized population of macrophages involved in neuroinflammatory processes. Overactivation of microglia cells in the cerebral cortex of FMS patients was recently demonstrated by Albrecht et al. using PET [65]. Inhibition of microglial activation by naltrexone or naloxone therefore has an anti-inflammatory effect through the decrease in production of neurotoxic chemicals [66, 67], which is suggested to contribute to its analgesic effect [25]. The ability to quantitatively assess microglial activation using nuclear methods may provide researchers and clinicians with the opportunity to use it as a biomarker for FMS activity and for measuring the effect of naltrexone.

There is no evidence from clinical trials that opioids are effective in treating FMS, and the EULAR guidelines discourage the use of opioid analgesics. Only tramadol (a weak opioid with mild SNRI activity), administered alone or together, with paracetamol is currently supported by the EULAR recommendations and was found to reduce pain by 30%. Generally, it is believed that only short-term use of opioids may be appropriate in carefully selected patients, particularly those with severe FMS [6].

### 3.8. Dopamine Receptor Agonists

Evidence indicating involvement of dopaminergic pathways in the pathophysiology of FMS has led to attempts to develop medications intervening in dopaminergic metabolism [68]. With most evidence about the benefit of dopaminergic agonists being sporadic, it is worth mentioning tergulide, which in a randomized, double-blind placebo-controlled trial, was found to improve FMS symptoms in a subgroup of patients with spinal stenosis (as opposed to the comparing all tergulide-assigned patients to the placebo group, where no significant improvement was found) [69]. Despite the EULAR recommendations for management of FMS from 2008, recommending the consideration of dopamine agonists [8], a meta-analysis conducted by Sommer et al. did not find them of proven benefit [70], and hence, they are not included in the revised 2016 EULAR recommendations [6].

### 3.9. Cannabinoids

There are two major active components in cannabinoids: tetrahydrocannabinol (THC) and cannabidiol (CBD). The former is the psychoactive component, which affects pain (as well as emotions) and works through CB1 and CB2 receptors. The latter has anti-inflammatory and analgesic traits. The THC:CBD therefore determines the product's overall effect [71]. CB1 cannabinoid receptors are found predominantly in the CNS and peripheral nervous system. Their agonists act along sensory pathways as modulators of pain [72]. With regard to the complex function of the endocannabinoid system in pain modulation, FMS is hypothesized to be induced, among other factors, by a lack of endocannabinoid activity [73].

The main cannabinoids studied were nabilone and dronabinol, with conflicting results. Three randomized controlled studies have been published regarding cannabinoid treatment for FMS to date: Fiz et al. reported a significant relief in pain two hours after consumption [74]. Skrabek et al. reported a reduction in pain, as well as level of anxiety, and an improved quality of life when using nabilone in comparison with placebo [26]. Ware et al. found a moderate effect on insomnia when using nabilone versus amitriptyline but no proven effect on pain or general quality of life [27].

A systematic review by Walitt et al. concluded that no convincing evidence suggests that nabilone is useful in treating people with FMS [28]. A work by Weber et al. showed dronabinol significantly reduce pain and depressive symptoms in FMS patients with neuropathic pain [29].

van de Donk et al. conducted a randomized, placebo-controlled trial exploring the analgesic effect of different cannabis varieties (with different THC:CBD contents) on fibromyalgia patients. They found that THC-containing products increased the pain threshold, whereas CBD-containing products increased plasma-THC levels but diminished THC analgesic effect [75]. This study demonstrated the complex effect of cannabinoids and the endocannabinoid system on chronic pain.

A prospective observational study recently published by Sagy et al. followed a relatively large cohort of FMS patients (*n* = 367) for a period of six months. The researchers showed a significant reduction in average pain intensity, sleep disturbance, and depression-related symptoms [76].

Cannabinoids have been offered by the Canadian guidelines for the management of FMS as a therapeutic option for FMS patients with prominent sleep abnormalities [10]. However, more controlled studies are needed to clarify the role of cannabinoids in this syndrome. Furthermore, research is called for focusing on the effects of various cannabinoids (as well as their combinations) on the basic neurophysiological aspects of FMS such as altered CNS connectivity patterns.

Manipulating the endocannabinoid system is gradually emerging as another fascinating strategy for treating pain [77]. Endocannabinoids such as anandamide are metabolized by specific enzymes including fatty acid amide hydrolase (FAAH) and monacylglycerol lipase (MAGL), and agents capable of inhibiting these enzymes are being tested as novel analgesic targets [78]. Future research into the clinical utility of endocannabinoid metabolism manipulation in FMS is expected.

### 3.10. NMDA Antagonists

Glutamate is the most abundant excitatory neurotransmitter in the nervous system. Central sensitization of pain transmission pathways is associated with hyperexcitability of the glutamatergic system, which leads symptoms observed in persons suffering from chronic pain [79].

The N-methyl-D-aspartate (NMDA) receptors are one of three subgroups of glutamate receptors. Activated by a variety of agonists, including substance P and neurokinin, it is known to be involved in the pathogenesis of central sensitization [80], a trait for which efforts were made to develop NMDA antagonists as therapeutic options for FMS, as well as other disorders resulting from central sensitization.

Ketamine, an NMDA antagonist, was found to reduce muscular and referred pain in FMS patients [30]. Memantine, another receptor antagonist, was suggested to be useful because of its ability to reduce neurotoxicity caused by high levels of glutamate found in different brain areas of FMS patients [31, 32]. These high glutamate levels were found to be related to the severity of FMS symptoms [81]. A double-blind, randomized-controlled trial published in 2014 found memantine to achieve a significant reduction in pain [33], with another hypothesis suggesting the combined use of pregabalin and memantine to concomitantly affect voltage-gated calcium channels and NMDA receptors, as a possible therapeutic approach [82]. A recent meta-analysis of 15 studies regarding the benefit of memantine in treating chronic pain (either neuropathic or FMS) concluded that the current evidence regarding memantine for chronic pain is limited and reported an increase in dizziness as a side effect of the medication [34].

## 4. Novel Therapeutic Approaches

NYX-2925 ((2S,3R)-3-hydroxy-2-((R)-5-isobutyryl-1-oxo-2,5-diazaspiro[3,4]octan-2-yl)butanamide) is a new NMDA receptor modulator which was shown by Khan et al. to affect NMDA receptor synaptic plasticity. This finding led to the hypothesis that it would be effective in NMDA receptor-associated CNS disorders [83]. As demonstrated by Ghoreishi-Haack et al. NYX-2925 was shown to induce efficient analgesia in rat models of neuropathic pain [35]. The first in human phase I trial recently published checked the safety, tolerability, and pharmacokinetics of the molecule in 84 healthy volunteers. NYX-2925 was shown to be safe, well tolerated, and to cross the blood-brain barrier. These promising findings support further clinical development of the drug as an agent for treating chronic pain conditions, such as diabetic neuropathy and FMS [36].

## 5. Conclusions and Expert Opinion

Chronic pain in general and FMS in particular continue to be an unmet challenge, while great progress has been made over recent years in the field of rheumatology in controlling the broad spectrum of inflammatory and autoimmune disorders, those clinical entities such as FMS which are centered on pain amplification within the CNS continue to baffle both clinicians and researchers alike due to their complexity. Thus, future breakthrough in the field of FMS will clearly necessitate a truly eclectic and multidisciplinary approach. Notably, while we may yet see in the future some incremental progress in the development of novel agents from the families currently in use, e.g., novel SNRIs or gabapentinoids, it does not seem likely that major breakthroughs will be found in these pathways.

### 5.1. Expert Opinion

When attempting to tackle such challenges, it appears useful to draw upon the experience and innovation accrued in other, parallel fields of medicine dealing with comparably complex CNS disorders. One example of such an initiative is the Research Domain Criteria (RDoC) of the NIAMH, which attempts to create an integrative framework for the research, classification, and future treatment of mental disorders, while incorporating evidence ranging from genomics through circuits, with the goal of eventually developing true precision treatments [84]. In the current point at time, while significant progress is being made into understanding connectivity patterns of FMS on the one hand, understanding the genetic underpinnings on the other hand, it seems self-evident that a comparable approach will need to be adopted in the study of chronic pain conditions such as FMS as well. Once such an integrative matrix is put in place, future clinicians will hopefully be able to treat specific patients based on their individual genetic (and pharmcogenetic) characteristics, together with an individualized analysis of connectivity patterns (notably, initial research in this direction is already underway, such as the results of Schmidt-Wilcke showing that decreased connectivity of brainstem pain-inhibitory centers can predict SNRI-responsiveness [85].

Another vastly understudied area to date, which would appear to hold great promise regarding the treatment of FMS, is the endocannabinoid system with all its diverse ramifications (as alluded to the above). We are currently on the brink of diving into this complex system and further research aimed at understanding the effects and interplay of large numbers of cannabinoids (phytocannbinoids as well as endocannabinoids, terpenes, etc.), their receptors, and their enzymatic control; Moreover, once we start understanding how these agents impact on the connectivity patterns mentioned above, we will truly be on the way towards rational utilization of cannabinoids for the treatment of FMS. The clinical development of high-quality medications, with precise delivery of measured quantities of cannabinoid agents such as THC and CBD (either through inhalation or otherwise) is a practical pre-requisite for future progress in this field, as is increased familiarity on the part of physicians.

Another field in which FMS treatment may find paths to success is related to the emerging field of neuroinflammation, as mentioned above in the context of the story of naltrexone. Just while the opioid epidemic is moving further into the focus of public attention, and it has become obvious that indiscriminate use of opioids is much more of a problem than a solution for chronic pain, and it will become ever more crucial to figure out the role played by opioid receptors (as well as by the endogenous opioids) in the maintenance of chronic pain and in the indication of opioid-induced hyperalgesia, as well as in the interplay with neuroinflammation (e.g., glial cell activation). Future treatments may target these pathways such as in the development of more specific antagonists or through the novel anti-inflammatory agents.

Lastly, it is always important to state that managing FMS is about more than just pharmacological therapy. As many patients and physicians have come to understand (and as reflected in the current guidelines) treating FMS will most probably continue to strive to combine pharmacotherapy with many nonpharmacological tools, ranging from exercise to neurofeedback and all that lie in between. Thus is the art of treating FMS.

## Figures and Tables

**Table 1:** Overview of effective pharmacological treatments for FM discussed in this review.

Drug	Mechanism	Effects on disease symptoms	Quality of evidence
Amitriptyline	Tricyclic antidepressant	Improvement in pain, fatigue, and sleep abnormalities.	Several randomized controlled trials, guideline recommended [8–10]

Duloxetine	Serotonin-noradrenaline reuptake inhibitor	Improvement in pain and depression.	Several randomized controlled trials, meta-analysis [11, 12]

Milnacipran	Serotonin-noradrenaline reuptake inhibitor	Improvement in pain and fatigue.	Several randomized controlled trials, meta-analysis [11, 12]

Reboxetine	Selective noradrenaline reuptake inhibitor	Improvement in pain.	Mostly case reports [13]

Esreboxetine	Selective noradrenaline reuptake inhibitor	Improvement in pain and fatigue.	Randomized controlled trial [14]

Citalopram, escitalopram, fluoxetine, paroxetine	Selective serotonin reuptake inhibitors	Improvement in pain and depression.	Randomized controlled trials, meta-analysis, guideline recommended [8–10, 12, 15, 16]

Cyclobenzaprine	5-HT2 receptor blocker	Moderately improves sleep, mild improvement in pain. Development stopped due to low efficacy.	Randomized controlled trial [17–19]

Pregabalin, gabapentin	Gabapentinoid	Improvement in pain, fatigue, and sleep abnormalities.	Randomized controlled trials, guideline recommended [8–10, 15, 20–22]

Lacosamide	Gabapentinoid	Effective in animal models. No clear evidence in FMS.	Randomized controlled trial [23]

Naltrexone	Opioid receptor antagonist, TLR-4 antagonist	Improvement in pain in depression.	Randomized controlled trial [24, 25]

Tramadol	Opioid with SNRI activity	Improvement in pain. Mostly for severe symptoms and short duration, see text.	Guideline recommended [6]

Nabilone	Cannabinoid	Improvement in pain and anxiety. Conflicting results, see text.	Randomized controlled trials, meta-analysis [26–28]

Dronabinol	Cannabinoid	Improvement in pain and depression. Conflicting results, see text.	Randomized controlled trials, meta-analysis [29]

Ketamine	NMDA antagonist	Improvement in referred pain.	Clinical trial, animal models [30]

Memantine	NMDA antagonist	Improvement in pain, conflicting evidence, see text.	Randomized controlled trials, meta-analysis [31–34]

NYX-2925	NMDA receptor modulator	Improvement in pain.	Animal models, pending clinical trials in humans [35, 36]

## Additional Points

*Highlights.* (i) Fibromyalgia is a pain disorder associated with comorbid somatic and psychological disorders. Its pathogenetic basis is complex but involves oversensitization and decreased conditioned pain modulation (CPM), as well as increased connectivity between different brain areas involved in pain modulation. (ii) The complexity of central mechanisms of chronic pain makes the pharmacologic therapy of fibromyalgia complex. Currently, only three drugs, pregabalin (a gabapentinoid), duloxetine, and milnacipran (SNRIs), are FDA-approved. Many other were tried over the years, with varying efficacy. (iii) Real-life data suggest that most fibromyalgia patients are not compliant with long-term pharmacotherapy. On the other hand, many of them are erroneously treated with multiple ineffective combinations of drugs, which confer no pain relief. (iv) Current guidelines recommend combining drugs with complimentary modalities, such as exercise and CBT. (v) Although not FDA-approved for the fibromyalgia indication, current guidelines recommend the use of amitriptyline and SSRIs. There is no evidence from clinical trials that opioids are effective in treating fibromyalgia, and their use is discouraged. Naltrexone, an opioid receptor antagonist, was shown in several trials to improve fibromyalgia symptoms. (vi) The endocannabinoid system and its role in pain modulation have been extensively researched. The Canadian guidelines support the use of cannabinoids mostly for fibromyalgia patients with prominent sleep abnormalities. More research is needed in this field, which holds much promise. (vii) Fibromyalgia treatment is still an unmet challenge. Especially, as our knowledge of pain amplification in the CNS, brain connectivity patterns and the role of neuroinflammation in the pathogenesis grow. The future of developing therapeutic measures for complex pain syndromes, including fibromyalgia, will necessitate a multidisciplinary approach, combining understanding the patient's pharmacogenomics and central connectivity patterns.
